# Effect of Influenza-Induced Fever on Human Bioimpedance Values

**DOI:** 10.1371/journal.pone.0125301

**Published:** 2015-04-27

**Authors:** Elisabetta Marini, Roberto Buffa, Monica Contreras, Magda Magris, Glida Hidalgo, Wilmer Sanchez, Vanessa Ortiz, Maryluz Urbaez, Stefano Cabras, Martin J. Blaser, Maria G. Dominguez-Bello

**Affiliations:** 1 University of Cagliari, Cagliari, Italy; 2 Venezuelan Institute of Scientific Research (IVIC), Caracas, Venezuela; 3 Amazonic Center for Research and Control of Tropical Diseases (CAICET), Puerto Ayacucho, Amazonas, Venezuela; 4 Foundation Center for Studies on Growth and Development of the Venezuelan Population, (Fundacredesa), Caracas, Venezuela; 5 Popular Ministry of Health, Caracas, Venezuela; 6 University Carlos III of Madrid, Madrid, Spain; 7 New York University School of Medicine, New York, New York, United States of America; 8 University of Puerto Rico, San Juan, Puerto Rico, United States of America; University of Palermo, ITALY

## Abstract

**Background and Aims:**

Bioelectrical impedance analysis (BIA) is a widely used technique to assess body composition and nutritional status. While bioelectrical values are affected by diverse variables, there has been little research on validation of BIA in acute illness, especially to understand prognostic significance. Here we report the use of BIA in acute febrile states induced by influenza.

**Methods:**

Bioimpedance studies were conducted during an H1N1 influenza A outbreak in Venezuelan Amerindian villages from the Amazonas. Measurements were performed on 52 subjects between 1 and 40 years of age, and 7 children were re-examined after starting Oseltamivir treatment. Bioelectrical Impedance Vector Analysis (BIVA) and permutation tests were applied.

**Results:**

For the entire sample, febrile individuals showed a tendency toward greater reactance (p=0.058) and phase angle (p=0.037) than afebrile individuals, while resistance and impedance were similar in the two groups. Individuals with repeated measurements showed significant differences in bioimpedance values associated with fever, including increased reactance (p<0.001) and phase angle (p=0.007), and decreased resistance (p=0.007) and impedance (p<0.001).

**Conclusions:**

There are bioelectrical variations induced by influenza that can be related to dehydration, with lower extracellular to intracellular water ratio in febrile individuals, or a direct thermal effect. Caution is recommended when interpreting bioimpedance results in febrile states.

## Introduction

Standard international criteria for the measurement of body composition using BIA indicate that values are affected by body position, consumption of food and beverages, recent physical activity, and ambient air and skin temperature [1]. Although minor variations of measurement conditions can be tolerated, the use of BIA in acute illnesses needs further validation, especially in febrile states, to understand its prognostic significance [2].

In early 2009, an outbreak of H1N1 influenza A started in Mexico and expanded worldwide [3]. In October 2009, while conducting morphometric anthropological and microbiological examinations of Amerindians in Amazonas State of Venezuela, we noted that many (52%) of the 52 studied persons in the *shabonos* of Platanal village had temperature ≥ 37.1°C. In the context of widespread upper respiratory symptoms, we recognized that H1N1 influenza A had come to this isolated population, which later was confirmed by the Venezuelan Ministry of Health. To mitigate the extent of the disease, medical personnel treated all persons with Oseltamivir. In this work we report the effect of the acute febrile state caused by epidemic influenza on bioimpedance measurements.

## Subjects and Methods

### Study population and measurements

Platanal/Mahecoto is a village of about 250 inhabitants living in a very remote region of the Venezuelan Amazon, with restricted access. There is a mission restricted to the presence of one priest, and a medical post that received young MDs doing their rural practice. People live traditional lifestyle, are hunter-gatherers, and live in houses built from thatch or of mud bricks on dirt floors.

Bioimpedance studies were conducted on 52 subjects (33 males and 19 females) between 1 and 40 years of age, under protocols and written consent approved by bioethical committees from Venezuelan Institute of Scientific Research (IVIC) (DIR 0229/10) and Amazonic Center for Research and Control of Tropical Diseases (CAICET) (N° DG-092-12). The written consent was signed by the chief of the village and by individual participants or mothers on behalf of their minors/children enrolled in the study.

Seven children (6 males and 1 females) were re-examined 24 h after starting Oseltamivir treatment. Body temperature was obtained with a “gun thermometer” from forehead measurements. Height was measured by a portable anthropometer. Resistance (R, Ω) and reactance (Xc, Ω) were measured with a single-frequency impedance analyzer (Quantum I, RJL Systems, Clinton Township, Michigan, USA) with an operating frequency of 50 kHz at 800 μA [1]. Whole body impedance measurements were obtained with the outer and inner electrodes on the right hand and foot [1].

### Bioelectrical impedance analyses

The phase angle (Φ, degrees) was calculated using the equation: Φ = arctan (Xc/R). Impedance (Z, Ω) was calculated with the equation: Z = (R^2^ + Xc^2^)^0.5^. Bioelectrical Impedance Vector Analysis (BIVA) was performed [4], allowing a synthetic representation of bioelectrical variability. Reactance reflects the capacitance produced by cell membranes of soft tissues, and is positively related with body cell mass [4], while resistance is negatively related with the proportion body water [4], through which the current flows. Impedance is mainly affected by resistance values. Phase angle is influenced by both reactance and resistance; it is positively related to body cell mass [4] and negatively related to the extracellular/intracellular water (ECW/ICW) ratio [5]. The analysis is based on the projection of values normalized by body height (H, in meters, m) in the Cartesian plane that is defined by the R/H and Xc/H axes (R/Xc graph), in which tolerance ellipses represent the bivariate percentiles of a reference population. Different regions of the ellipses have specific meanings in terms of body composition. Dehydrated individuals lie toward the upper pole of the ellipse, those with edema toward the lower pole; individuals characterized by low soft tissue mass lie on the right side of the ellipse, those with high soft tissue mass on the left side.

R/H, Xc/H, phase and impedance were standardized for sex and age using bioelectrical Italian standards [6,7]. The differences between the mean impedance vectors in febrile and normal individuals were assessed by Hotelling T^2^, and by permutation tests, a conservative statistical approach that is not based on the assumption of normality. Permuted mean differences are 10000 mean differences calculated under an equal random of permutations for the label (f) and (wf). For each permutation we calculated a mean difference and the histogram shows such differences. The p-value is just the proportion of such 10000 differences larger than the observed one. The differences between the mean impedance vectors before and 24h after the first Oseltamivir dose were assessed with paired one-sample Hotelling’s T^2^ test, and by permutation tests. Analyses were performed using specific BIVA software and the R program (http://www.R-project.org).

## Results

Of the 52 individuals considered for bioimpedance analysis, 27 had body temperature ≥ 37.1° (S1 Table).

H1N1 influenza was confirmed in the community (by real-time reverse transcriptase-polymerase chain reaction (rRT-PCR), determined by the Ministry of Health assigned laboratory), and people presented fever, headache, backache, and weakness. The epidemics source was a sport event the previous week, for which some Amerindians from the village travelled to the capital town of Puerto Ayacucho. Tamiflu was provided (was the recommended by WHO at the time, i.e: 5 days treatment of 75mg twice daily in adults and 3 mg/kg/dose twice daily in children) by the medical personnel.

**Fig 1:**
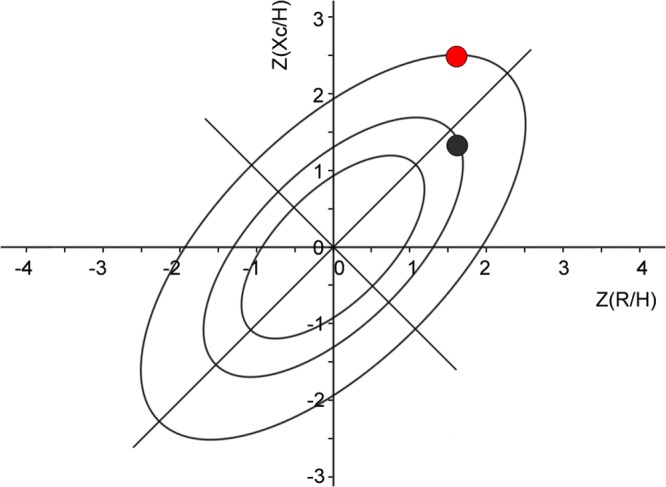
Mean bioelectrical values of subjects with fever (red; N = 27) and without (black; N = 25). R: resistance (Ohm); Xc: reactance (Ohm); H: height (m); Z(R/H) and Z(Xc/H): R/H and Xc/H standardized for sex and age using bioelectrical Italian standards.

**Fig 2:**
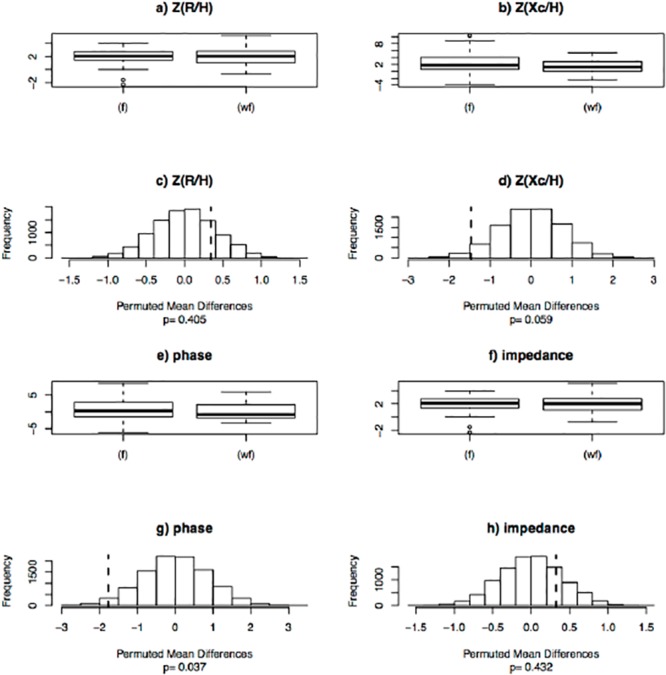
Permutation tests for the subjects with (f) or without (wf) fever. Sex and age observed standardized resistance, Z(R/H), and reactance, Z(Xc/H), box plots (**panels a, b**) and frequency distribution of permuted mean differences (**panels c, d**). Sex and age observed standardized phase and impedance box plots (**panels e, f**) and frequency distribution of permuted mean differences (**panels g, h**). The vertical dashed lines in the frequency graphs (**panels c, d, g and h**) show the corresponding observed mean difference. P-values for the corresponding permutation tests are reported under each histogram. R: resistance (Ohm); Xc: reactance (Ohm); H: height (m); phase: phase angle of the impedance vector (degrees); impedance: length of the impedance vector (Ohm).

The bioelectrical mean vector from the febrile individuals was located on the edge of the 95% tolerance ellipse, toward the dehydration region of the RXc graph. On the other hand, the location of the mean vector from afebrile individuals was inside the 75% tolerance ellipse, the same upper pole orientation (*p* = 0.40, Hotelling T^2^ test) (Fig 1). Using permutation tests, febrile individuals showed greater reactance (*p* = 0.059) and phase angle (*p* = 0.037) than afebrile individuals, while there were no significant differences for resistance (p = 0.405) and impedance (p = 0.432), between the two groups (Fig 2; S2 Table).

**Fig 3:**
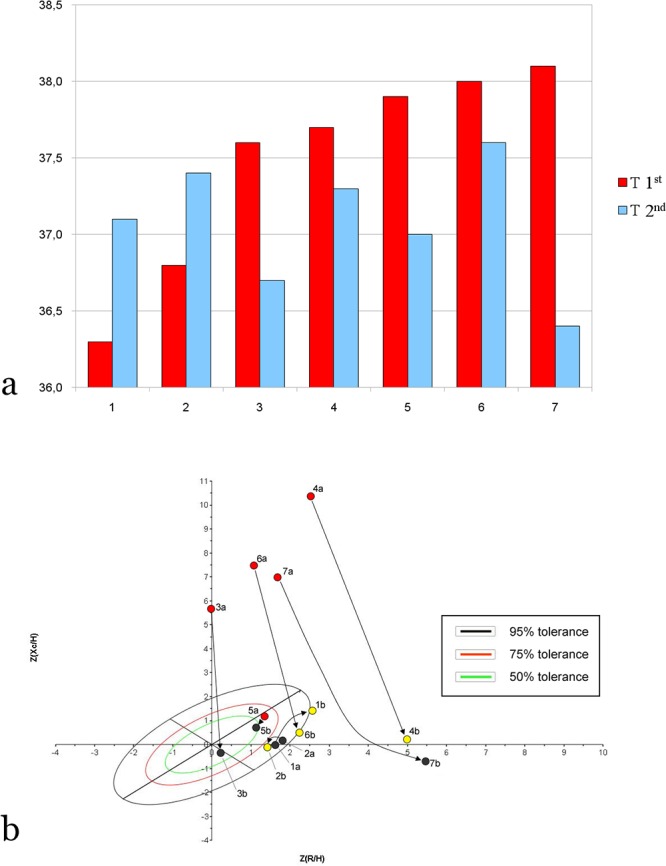
Changes in body temperature after 24h of Oseltamivir treatment, and bioelectrical impedance vector analysis (BIVA). **Panel a:** Body temperature in seven children at the time of initial BIVA (red bars) and then 24 hours after receiving the first dose of Oseltamivir (blue bars). "T 1^st^" and "T 2nd” refer to the corresponding temperatures. Children’s ages are: 1, 10 y; 2, 10 y; 3, 12 y; 4, 9 y; 5, 16 y; 6, 11 y; 7, 5 y. **Panel b:** BIVA (Z score = standardized data of paired data from children with or without fever (T≥37.5°C, red dots; 37.1°C≤ T<37.5°C, yellow dots; T≤37°C, black dots), with direction of arrows showing the vector migration from the initial measurement.

Paired bioimpedance measurements were obtained from seven children 24h after they started Oseltamivir treatment (S3 Table). Of these children, three had returned to normal temperature (≤37°C), one remained febrile (T > 37.5°C), and three had body temperature between 37.1°C and 37.4°C. Bioelectrical values changed significantly as temperature changed (p<0.001, paired one-sample Hotelling T^2^ test) (Fig 3). Using permutation tests, the fever of influenza was associated with increased reactance (*p*<0.001) and phase angle (*p* = 0.007) and with decreased resistance (*p* = 0.007) and impedance values (*p*<0.001).

## Discussion

BIVA has proven to be a very useful tool to determine the nutritional status of children and adults in remote populations [8–10]. In relation to more transculturated Venezuelan Amerindians from the ethnic group Guahibo, living in a community near a road South of Puerto Ayacucho [8] [10], the bioelectrical values of the Yanomami were generally more oriented toward the upper pole of the tolerance ellipses, i.e. in the region of dehydration. This pattern was especially marked in febrile individuals, who showed more dehydration, but we cannot discard that Yanomami who were afebrile at the time of measurement had mild cases of influenza, given the 2009 H1N1 high transmission rates in adults [11].

The effect of influenza was evidenced in the measurements before and after the treatment with Oseltamivir, since the antiviral is not expected to directly change hydration status in short time. The bioelectrical values of febrile states were far outside the tolerance ellipses, while fever remission induced a general normalization of bioelectrical values. The major effects of influenza-induced fever were related to increases of reactance and phase angle. There also was a lesser but significant variation toward lower resistance and impedance values.

The observed decreases in phase angle during fever remission could reflect loss of cell mass, but this is unlikely to occur within 24 h. A more likely interpretation of the high phase angle in febrile states is the lowering of ECW/ICW ratio [5], due to loss of extracellular water. Manifestations of influenza, including chills, sweating, vomiting, and diarrhea may affect short-term hydration state and bioelectrical measurements. In other acute illness, such as in classical dengue, patients show a reduction of resistance and reactance after the acute febrile phase of illness, during remission, which is consistent with the relative expansion normalization of extracellular water [12].

However, direct thermal effects of febrile states that alter the electrical conductivity of the biological tissues cannot be excluded. In fact, skin temperature can affect BIA measurements [1]. In particular, consistently with our results on individuals with repeated measurements, the provoked increases in skin temperature lower both resistance and impedance values [13].

By conducting these studies in the midst of an influenza outbreak in isolated peoples, we now provide evidence that a febrile illness increases phase angle and reactance, and to a lesser extent decreases resistance and impedance, with values tending to normalize following appropriate therapy. These bioelectrical changes could be related to variations of body hydration, with an apparent decrease of extracellular water. The results of this work warrant caution when interpreting bioimpedance in febrile states, highlight the importance of measuring body temperature before performing bioelectrical measurements, and in the case of febrile stages, assess nutritional status by other methods.

## Supporting Information

S1 Table Individual data on age, gender, height, resistance, reactance, and body temperature. Age in years; Gender: 1, males; 2, females; Height in cm; R (Resistance) and Xc (reactance) in Ohm; Temperature in degrees Celsius
(DOCX)

S2 Table Mean age, body temperature and standardized bioelectrical values in subjects with and without fever. Z(R/H): standardized R/H (resistance normalized by height, Ohm/m); Z(Xc/H): standardized Xc/H (reactance normalized by height, Ohm/m).
(DOCX)

S3 Table Bioelectrical values in seven subjects before and after receiving Oseltamivir treatment. R/H: resistance normalized by height (Ohm/m); Z(R/H): standardized R/H; Xc/H: reactance normalized by height (Ohm/m); Z(Xc/H): standardized Xc/H; Phase: phase angle of the impedance vector; Z/H: vector length normalized by height (Ohm/m); T: skin temperature (degrees Celsius); "1st" and "2nd” refer to the measurements at the time of initial BIVA and then 24 hours after receiving the first dose of Oseltamivir.
(DOCX)
