# Supplementary material for: Effect of Influenza-Induced Fever on Human Bioimpedance Values
Source: PLoS One. 2015 Apr 27;10(4):e0125301. doi: 10.1371/journal.pone.0125301 (PMC4410917; doi:10.1371/journal.pone.0125301)
Supplement: S1 Table — Age in years; Gender: 1, males; 2, females; Height in cm; R (Resistance) and Xc (reactance) in Ohm; Temperature in degrees Celsius (DOCX) [file pone.0125301.s001.docx]

| Age | Gender | Height | R | Xc | Temperature |
| --- | --- | --- | --- | --- | --- |
| 16 | 1 | 148 | 515 | 58 | 37.9 |
| 17 | 1 | 146.1 | 491 | 62 | 36.9 |
| 17 | 1 | 148.5 | 608 | 70 | 37.3 |
| 15 | 1 | 145.8 | 595 | 68 | 37.2 |
| 33 | 1 | 150.4 | 605 | 93 | 36.7 |
| 24 | 2 | 146.3 | 690 | 51 | 36.9 |
| 12 | 1 | 140.7 | 645 | 61 | 36.6 |
| 11 | 1 | 134.3 | 735 | 60 | 36.6 |
| 10 | 1 | 120.4 | 684 | 57 | 37.4 |
| 12 | 1 | 129.4 | 725 | 62 | 36.7 |
| 9 | 2 | 116.7 | 752 | 63 | 36.6 |
| 10 | 2 | 127.2 | 808 | 72 | 37.1 |
| 8 | 1 | 130.8 | 618 | 73 | 37.4 |
| 10 | 1 | 134.1 | 648 | 59 | 36.7 |
| 9 | 1 | 118.7 | 750 | 53 | 36.8 |
| 11 | 1 | 121.6 | 668 | 113 | 38.0 |
| 7 | 1 | 113.7 | 680 | 86 | 36.5 |
| 11 | 1 | 145.5 | 515 | 36 | 37.3 |
| 12 | 1 | 142.9 | 843 | 65 | 37.2 |
| 7 | 1 | 117.6 | 776 | 58 | 37.3 |
| 12 | 1 | 137.8 | 553 | 40 | 36.9 |
| 28 | 1 | 159 | 588 | 23 | 37.7 |
| 15 | 1 | 142.8 | 834 | 62 | 36.1 |
| 15 | 2 | 137.3 | 683 | 51 | 35.9 |
| 8 | 2 | 114.6 | 613 | 98 | 36.6 |
| 9 | 2 | 122 | 862 | 64 | 36.0 |
| 12 | 1 | 143.4 | 630 | 112 | 37.6 |
| 6 | 2 | 113.2 | 700 | 87 | 37.3 |
| 5 | 1 | 86 | 682 | 98 | 38.1 |
| 6 | 2 | 96.1 | 808 | 113 | 37.4 |
| 8 | 1 | 110.6 | 670 | 95 | 36.8 |
| 10 | 2 | 119.6 | 760 | 63 | 37.1 |
| 5 | 1 | 89.5 | 715 | 74 | 36.2 |
| 6 | 2 | 95.3 | 813 | 73 | 37.1 |
| 9 | 1 | 104.5 | 691 | 130 | 37.7 |
| 7 | 1 | 103.6 | 722 | 97 | 37.0 |
| 7 | 1 | 102.4 | 815 | 70 | 37.3 |
| 7 | 1 | 99.3 | 693 | 68 | 37.4 |
| 10 | 2 | 120.7 | 730 | 95 | 37.2 |
| 37 | 1 | 156.6 | 619 | 60 | 37.5 |
| 20 | 1 | 148.6 | 649 | 77 | 37.4 |
| 22 | 2 | 138.8 | 780 | 106 | 37.4 |
| 20 | 2 | 140 | 731 | 65 | 37.0 |
| 6 | 1 | 106.5 | 910 | 66 | 36.6 |
| 18 | 2 | 139 | 665 | 65 | 36.9 |
| 15 | 2 | 141 | 750 | 66 | 37.3 |
| 40 | 2 | 146.7 | 744 | 91 | 36.7 |
| 6 | 2 | 117.8 | 872 | 61 | 37.2 |
| 26 | 1 | 147.4 | 542 | 58 | 37.0 |
| 15 | 1 | 147.2 | 617 | 69 | 37.2 |
| 35 | 2 | 143.5 | 730 | 62 | 36.4 |
| 25 | 2 | 142 | 749 | 79 | 36.3 |
|  | | | | | |
