# Supplementary material for: Effect of Influenza-Induced Fever on Human Bioimpedance Values
Source: PLoS One. 2015 Apr 27;10(4):e0125301. doi: 10.1371/journal.pone.0125301 (PMC4410917; doi:10.1371/journal.pone.0125301)
Supplement: S2 Table — Z(R/H): standardized R/H (resistance normalized by height, Ohm/m); Z(Xc/H): standardized Xc/H (reactance normalized by height, Ohm/m). (DOCX) [file pone.0125301.s002.docx]

|  | Subjects without fever  (N=25) | Subjects with fever  (N=27) |
| --- | --- | --- |
|  | Mean + SD | Mean + SD |
| **Age** | 15.7 + 9.7 | 12.5 + 7.4 |
| **Temperature** | 36.6 + 0.3 | 37.4 + 0.3 |
| **Z(R/H)** | 1.87 + 1.40 | 1.86 + 1.48 |
| **Z(Xc/H)** | 1.43 + 2.01 | 2.46 + 3.32 |
| **Phase** | 0.19 + 2.69 | 0.96 + 3.49 |
| **Impedance** | 1.88 + 1.40 | 1.89 + 1.49 |
