# Supplementary material for: Effect of Influenza-Induced Fever on Human Bioimpedance Values
Source: PLoS One. 2015 Apr 27;10(4):e0125301. doi: 10.1371/journal.pone.0125301 (PMC4410917; doi:10.1371/journal.pone.0125301)
Supplement: S3 Table — R/H: resistance normalized by height (Ohm/m); Z(R/H): standardized R/H; Xc/H: reactance normalized by height (Ohm/m); Z(Xc/H): standardized Xc/H; Phase: phase angle of the impedance vector; Z/H: vector length normalized by height (Ohm/m); T: skin temperature (degrees Celsius); "1st" and "2nd” refer to the measurements at the time of initial BIVA and then 24 hours after receiving the first dose of Oseltamivir. (DOCX) [file pone.0125301.s003.docx]

|  | **R/H** | | **Z(R/H)** | | **Xc/H** | | **Z(Xc/H)** | | **Phase** | | **Z/H** | | **T** | |
| --- | --- | --- | --- | --- | --- | --- | --- | --- | --- | --- | --- | --- | --- | --- |
| **Subject (age)** | **1st** | **2nd** | **1st** | **2nd** | **1st** | **2nd** | **1st** | **2nd** | **1st** | **2nd** | **1st** | **2nd** | **1st** | **2nd** |
| 1 (10 y) | 580.2 | 635.2 | 1.62 | 2.57 | 48.0 | 56.6 | -0.01 | 1.43 | 4.7 | 5.1 | 637.0 | 637.7 | 36.3 | 37.1 |
| 2 (10 y) | 590.5 | 568.1 | 1.80 | 1.42 | 49.0 | 47.3 | 0.17 | -0.11 | 4.8 | 4.7 | 570.2 | 570.1 | 36.8 | 37.4 |
| 3 (12 y) | 439.3 | 453.3 | -0.01 | 0.23 | 78.1 | 41.8 | 5.68 | -0.36 | 10.1 | 5.3 | 446.2 | 455.2 | 37.6 | 36.7 |
| 4 (9 y) | 661.2 | 791.4 | 2.53 | 4.99 | 124.4 | 53.6 | 10.34 | 0.23 | 10.7 | 3.9 | 672.8 | 794.2 | 37.7 | 37.3 |
| 5 (16 y) | 348.0 | 338.5 | 1.35 | 1.13 | 39.2 | 35.8 | 1.17 | 0.70 | 6,4 | 6.0 | 350.2 | 340.4 | 37.9 | 37.0 |
| 6 (11 y) | 549.3 | 616.8 | 1.09 | 2.25 | 92.9 | 51.0 | 7.49 | 0.50 | 9.6 | 4.7 | 557.1 | 618.9 | 38.0 | 37.6 |
| 7 (5 y) | 793.0 | 1064.0 | 1.68 | 5.44 | 114.0 | 52.3 | 6.99 | -0.71 | 8.2 | 2.8 | 801.2 | 1065.2 | 38.1 | 36.4 |
